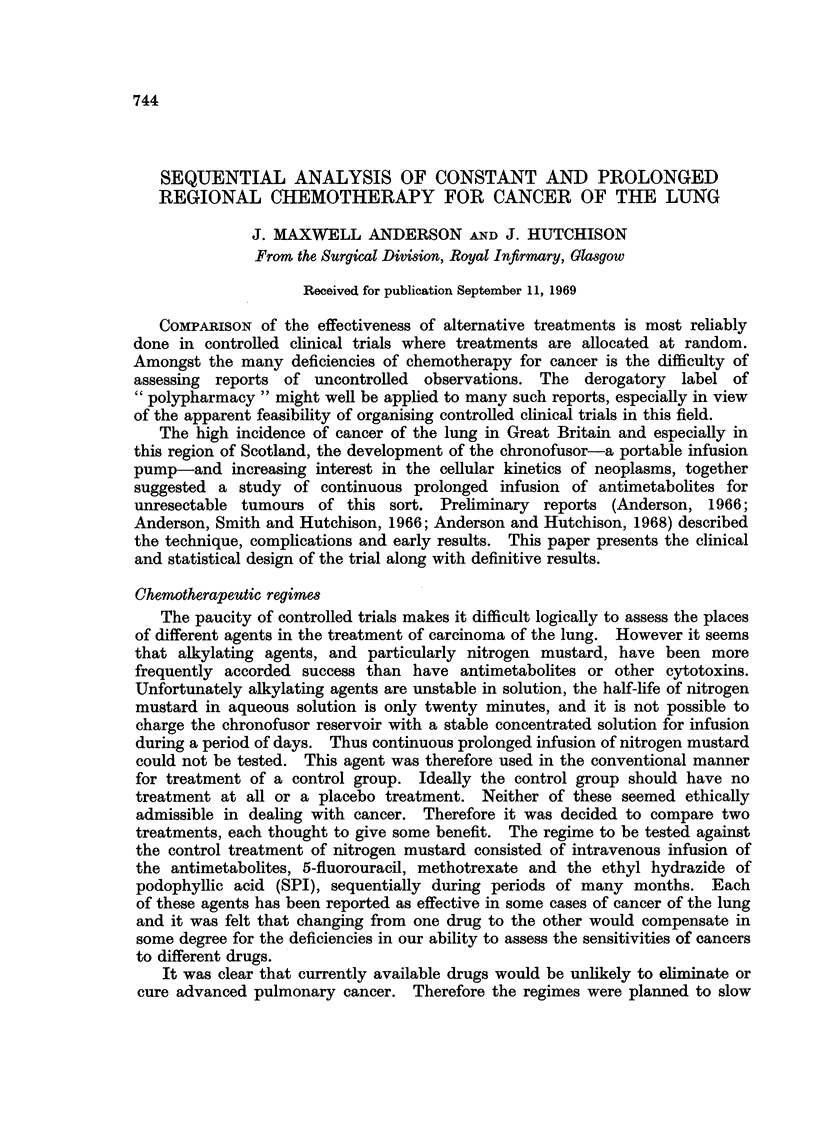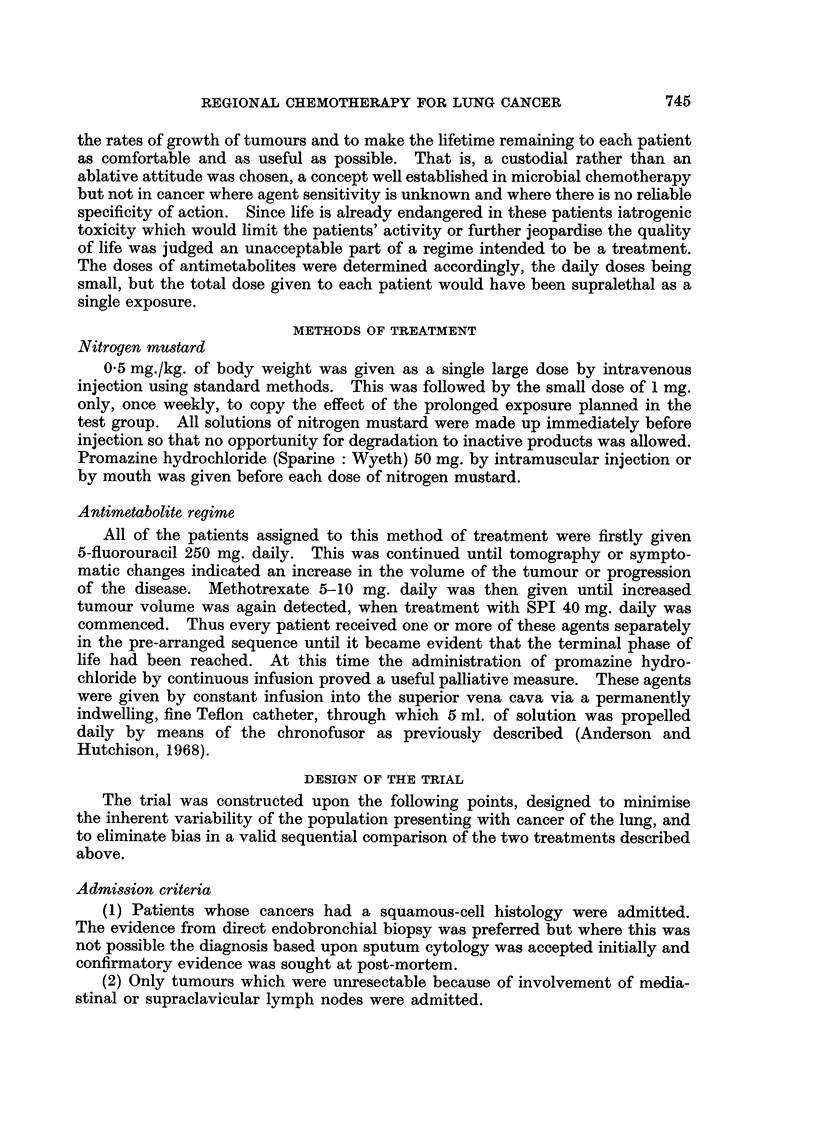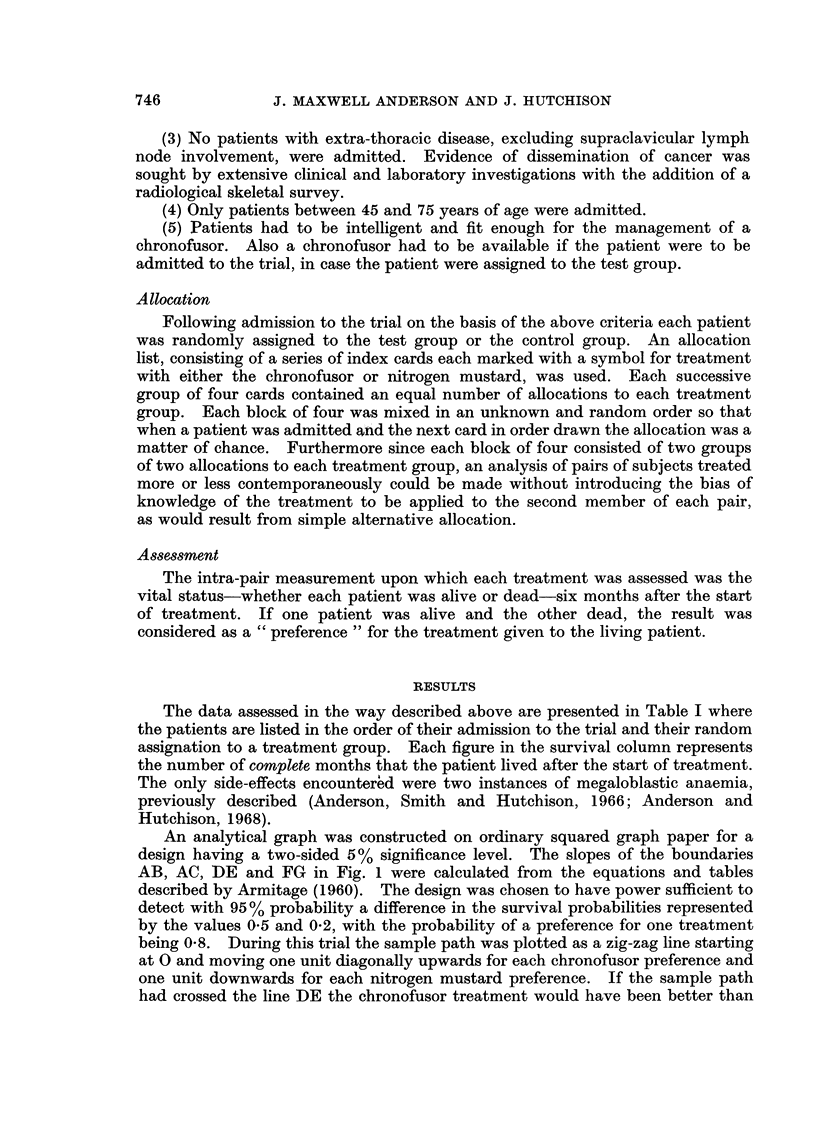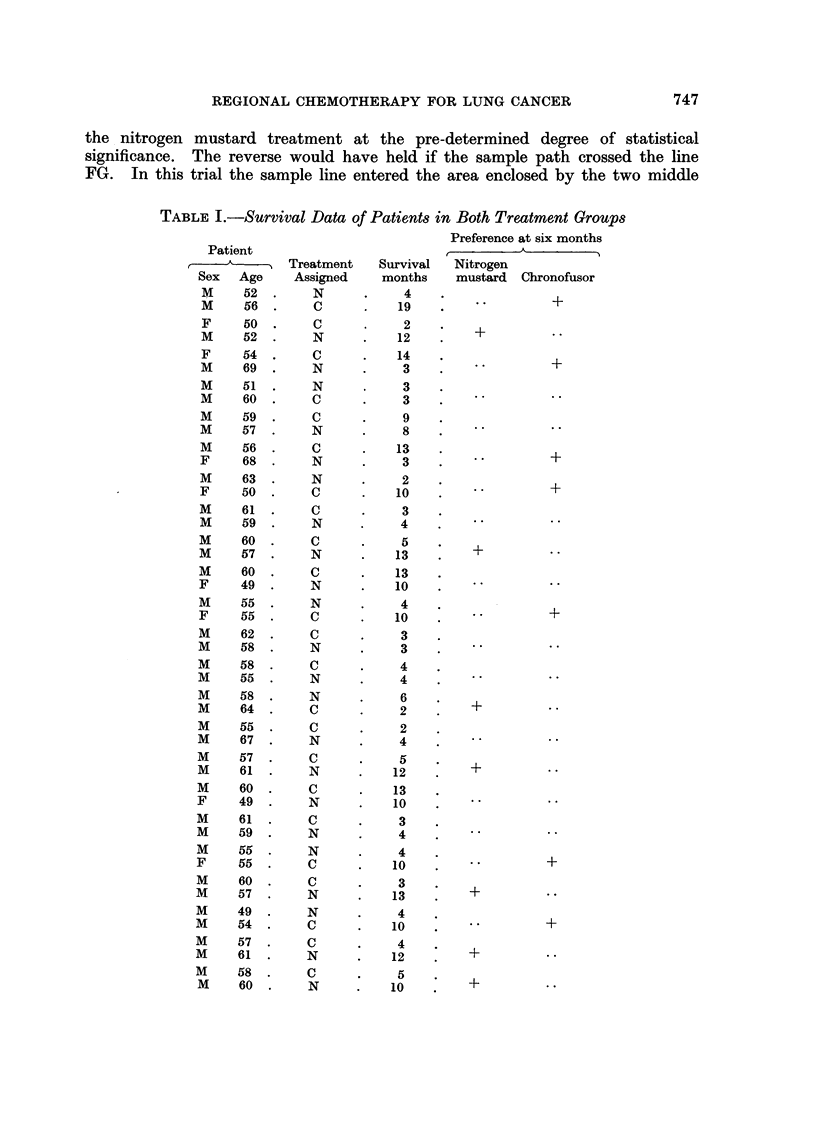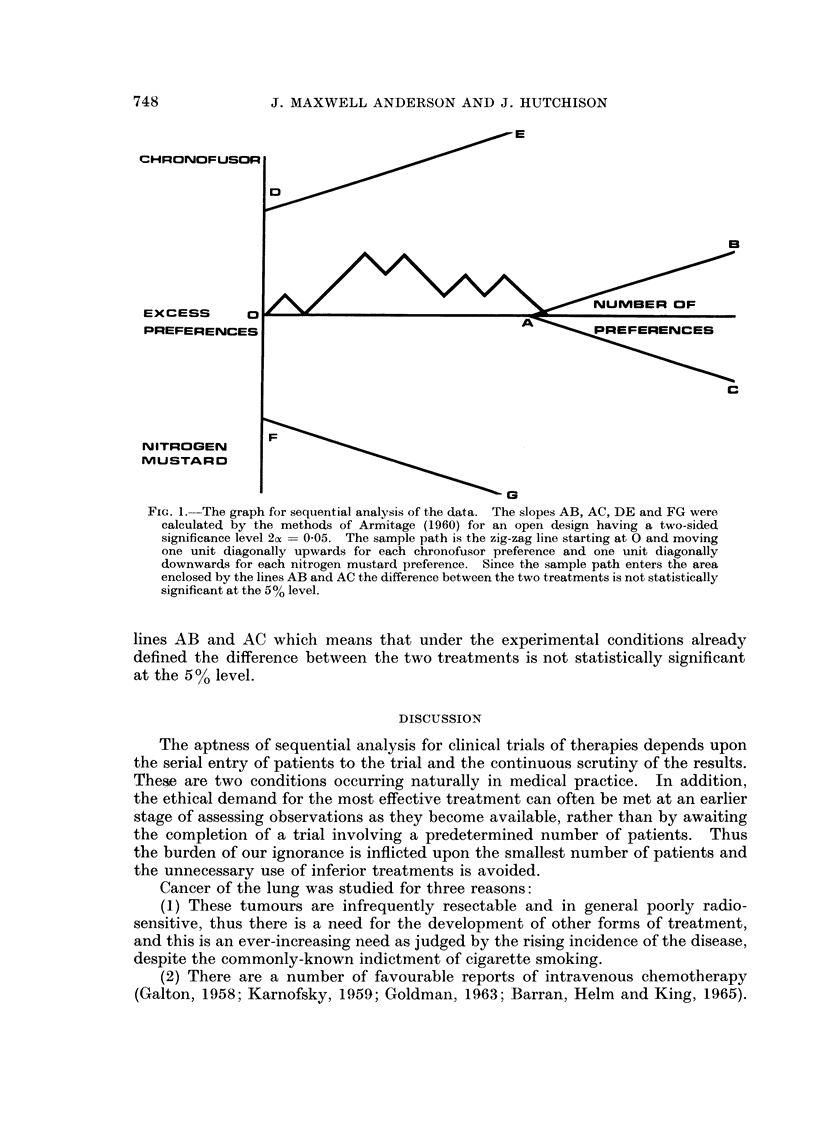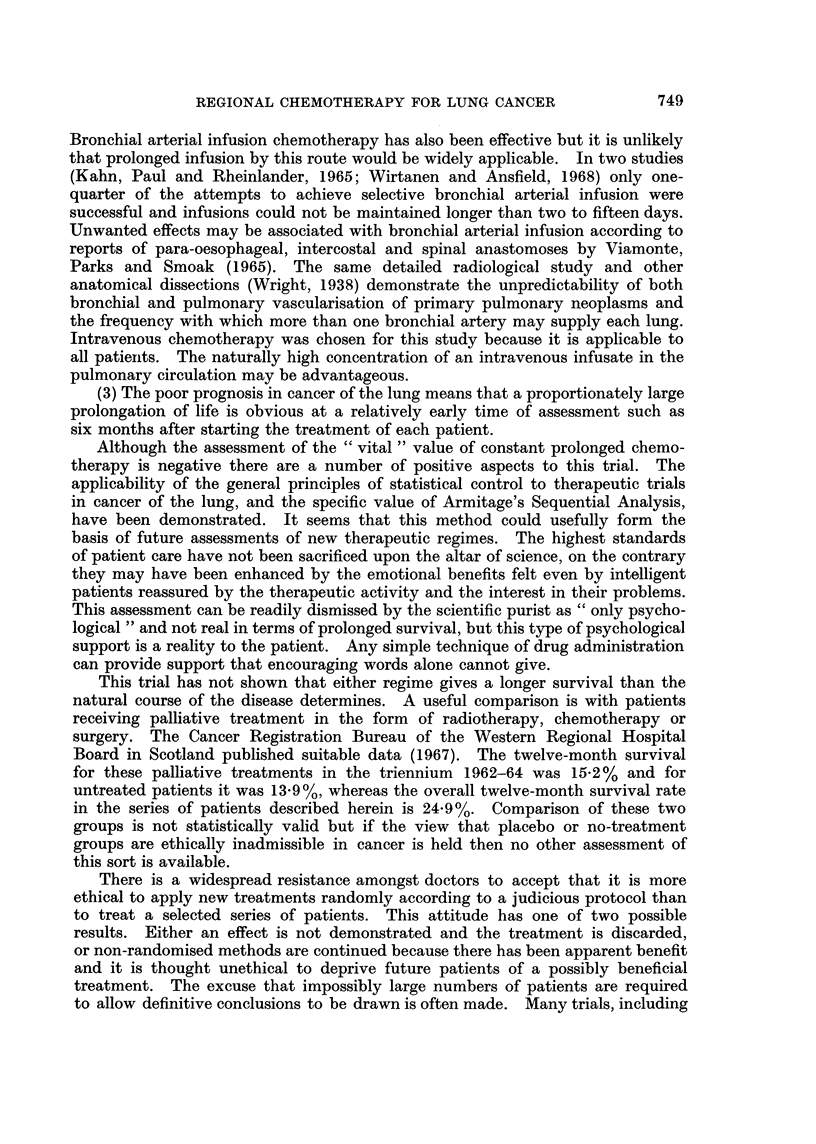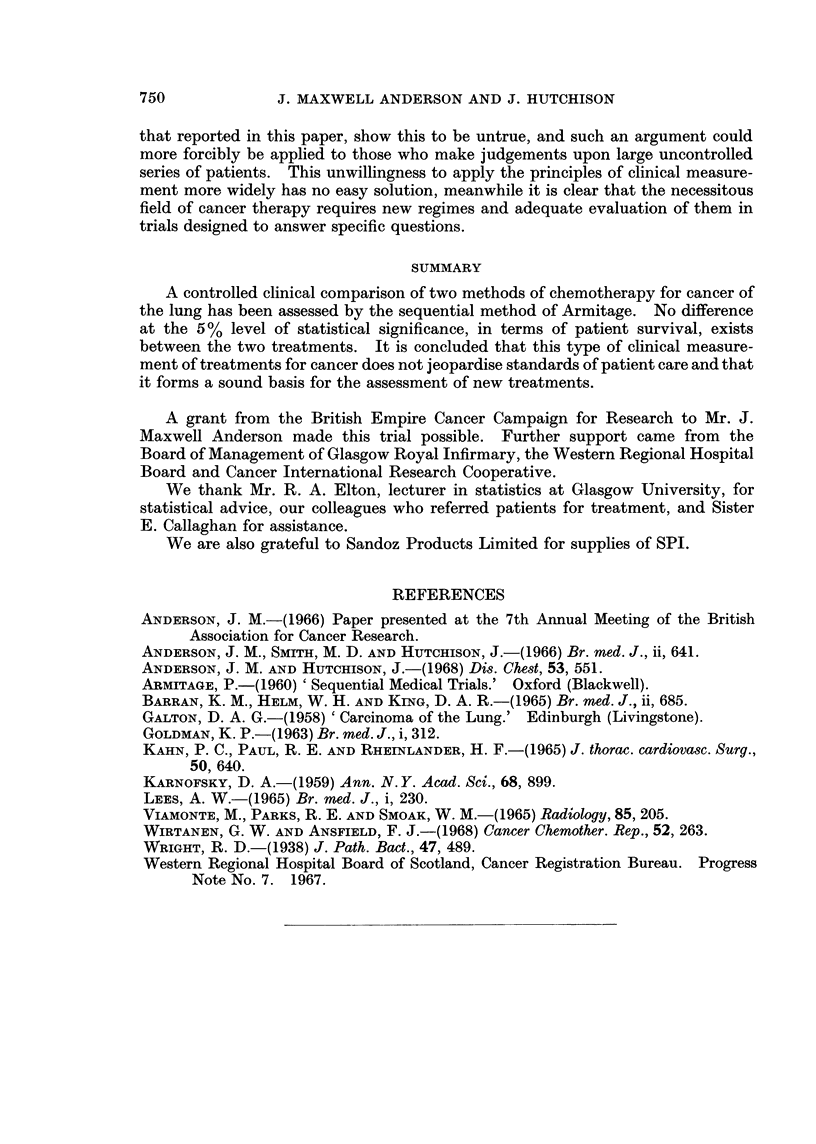# Sequential analysis of constant and prolonged regional chemotherapy for cancer of the lung.

**DOI:** 10.1038/bjc.1969.91

**Published:** 1969-12

**Authors:** J. M. Anderson, J. Hutchison


### SEQUENTIAL ANALYSIS OF CONSTANT AND PROLONGED REGIONAL CHEMOTHERAPY FOR CANCER OF THE LUNG

J. MAXWELL ANDERSON AND J. HUTCHISON
*From the Surgical Division, Royal Infirmary, Glasgow*

Received for publication September 11, 1969

COMPARISON of the effectiveness of alternative treatments is most reliably done in controlled clinical trials where treatments are allocated at random. Amongst the many deficiencies of chemotherapy for cancer is the difficulty of assessing reports of uncontrolled observations. The derogatory label of " polypharmacy " might well be applied to many such reports, especially in view of the apparent feasibility of organising controlled clinical trials in this field.

The high incidence of cancer of the lung in Great Britain and especially in this region of Scotland, the development of the chronofusor—a portable infusion pump—and increasing interest in the cellular kinetics of neoplasms, together suggested a study of continuous prolonged infusion of antimetabolites for unresectable tumours of this sort. Preliminary reports (Anderson, 1966; Anderson, Smith and Hutchison, 1966; Anderson and Hutchison, 1968) described the technique, complications and early results. This paper presents the clinical and statistical design of the trial along with definitive results.

*Chemotherapeutic regimes*

The paucity of controlled trials makes it difficult logically to assess the places of different agents in the treatment of carcinoma of the lung. However it seems that alkylating agents, and particularly nitrogen mustard, have been more frequently accorded success than have antimetabolites or other cytotoxins. Unfortunately alkylating agents are unstable in solution, the half-life of nitrogen mustard in aqueous solution is only twenty minutes, and it is not possible to charge the chronofusor reservoir with a stable concentrated solution for infusion during a period of days. Thus continuous prolonged infusion of nitrogen mustard could not be tested. This agent was therefore used in the conventional manner for treatment of a control group. Ideally the control group should have no treatment at all or a placebo treatment. Neither of these seemed ethically admissible in dealing with cancer. Therefore it was decided to compare two treatments, each thought to give some benefit. The regime to be tested against the control treatment of nitrogen mustard consisted of intravenous infusion of the antimetabolites, 5-fluorouracil, methotrexate and the ethyl hydrazide of podophyllic acid (SPI), sequentially during periods of many months. Each of these agents has been reported as effective in some cases of cancer of the lung and it was felt that changing from one drug to the other would compensate in some degree for the deficiencies in our ability to assess the sensitivities of cancers to different drugs.

It was clear that currently available drugs would be unlikely to eliminate or cure advanced pulmonary cancer. Therefore the regimes were planned to slow the rates of growth of tumours and to make the lifetime remaining to each patient as comfortable and as useful as possible. That is, a custodial rather than an ablative attitude was chosen, a concept well established in microbial chemotherapy but not in cancer where agent sensitivity is unknown and where there is no reliable specificity of action. Since life is already endangered in these patients iatrogenic toxicity which would limit the patients' activity or further jeopardise the quality of life was judged an unacceptable part of a regime intended to be a treatment. The doses of antimetabolites were determined accordingly, the daily doses being small, but the total dose given to each patient would have been supralethal as a single exposure.

### METHODS OF TREATMENT

### *Nitrogen mustard*

0·5 mg./kg. of body weight was given as a single large dose by intravenous injection using standard methods. This was followed by the small dose of 1 mg. only, once weekly, to copy the effect of the prolonged exposure planned in the test group. All solutions of nitrogen mustard were made up immediately before injection so that no opportunity for degradation to inactive products was allowed. Promazine hydrochloride (Sparine : Wyeth) 50 mg. by intramuscular injection or by mouth was given before each dose of nitrogen mustard.

### *Antimetabolite regime*

All of the patients assigned to this method of treatment were firstly given 5-fluorouracil 250 mg. daily. This was continued until tomography or symptomatic changes indicated an increase in the volume of the tumour or progression of the disease. Methotrexate 5–10 mg. daily was then given until increased tumour volume was again detected, when treatment with SPI 40 mg. daily was commenced. Thus every patient received one or more of these agents separately in the pre-arranged sequence until it became evident that the terminal phase of life had been reached. At this time the administration of promazine hydrochloride by continuous infusion proved a useful palliative measure. These agents were given by constant infusion into the superior vena cava via a permanently indwelling, fine Teflon catheter, through which 5 ml. of solution was propelled daily by means of the chronofusor as previously described (Anderson and Hutchison, 1968).

### DESIGN OF THE TRIAL

The trial was constructed upon the following points, designed to minimise the inherent variability of the population presenting with cancer of the lung, and to eliminate bias in a valid sequential comparison of the two treatments described above.

### *Admission criteria*

(1) Patients whose cancers had a squamous-cell histology were admitted. The evidence from direct endobronchial biopsy was preferred but where this was not possible the diagnosis based upon sputum cytology was accepted initially and confirmatory evidence was sought at post-mortem.

(2) Only tumours which were unresectable because of involvement of mediastinal or supraclavicular lymph nodes were admitted.

(3) No patients with extra-thoracic disease, excluding supraclavicular lymph node involvement, were admitted. Evidence of dissemination of cancer was sought by extensive clinical and laboratory investigations with the addition of a radiological skeletal survey.

(4) Only patients between 45 and 75 years of age were admitted.

(5) Patients had to be intelligent and fit enough for the management of a chronofusor. Also a chronofusor had to be available if the patient were to be admitted to the trial, in case the patient were assigned to the test group.

*Allocation*

Following admission to the trial on the basis of the above criteria each patient was randomly assigned to the test group or the control group. An allocation list, consisting of a series of index cards each marked with a symbol for treatment with either the chronofusor or nitrogen mustard, was used. Each successive group of four cards contained an equal number of allocations to each treatment group. Each block of four was mixed in an unknown and random order so that when a patient was admitted and the next card in order drawn the allocation was a matter of chance. Furthermore since each block of four consisted of two groups of two allocations to each treatment group, an analysis of pairs of subjects treated more or less contemporaneously could be made without introducing the bias of knowledge of the treatment to be applied to the second member of each pair, as would result from simple alternative allocation.

*Assessment*

The intra-pair measurement upon which each treatment was assessed was the vital status—whether each patient was alive or dead—six months after the start of treatment. If one patient was alive and the other dead, the result was considered as a " preference " for the treatment given to the living patient.

### RESULTS

The data assessed in the way described above are presented in Table I where the patients are listed in the order of their admission to the trial and their random assignation to a treatment group. Each figure in the survival column represents the number of *complete* months that the patient lived after the start of treatment. The only side-effects encountered were two instances of megaloblastic anaemia, previously described (Anderson, Smith and Hutchison, 1966; Anderson and Hutchison, 1968).

An analytical graph was constructed on ordinary squared graph paper for a design having a two-sided 5% significance level. The slopes of the boundaries AB, AC, DE and FG in Fig. 1 were calculated from the equations and tables described by Armitage (1960). The design was chosen to have power sufficient to detect with 95% probability a difference in the survival probabilities represented by the values 0·5 and 0·2, with the probability of a preference for one treatment being 0·8. During this trial the sample path was plotted as a zig-zag line starting at O and moving one unit diagonally upwards for each chronofusor preference and one unit downwards for each nitrogen mustard preference. If the sample path had crossed the line DE the chronofusor treatment would have been better than the nitrogen mustard treatment at the pre-determined degree of statistical significance. The reverse would have held if the sample path crossed the line FG. In this trial the sample line entered the area enclosed by the two middle

TABLE I.—*Survival Data of Patients in Both Treatment Groups*

| Patient | | Treatment | Survival | Preference at six months | |
|---|---|---|---|---|---|
| Sex | Age | Assigned | months | Nitrogen mustard | Chronofusor |
| M | 52 | N | 4 | | |
| M | 56 | C | 19 | .. | + |
| F | 50 | C | 2 | | |
| M | 52 | N | 12 | + | .. |
| F | 54 | C | 14 | | |
| M | 69 | N | 3 | .. | + |
| M | 51 | N | 3 | | |
| M | 60 | C | 3 | .. | .. |
| M | 59 | C | 9 | | |
| M | 57 | N | 8 | .. | .. |
| M | 56 | C | 13 | | |
| F | 68 | N | 3 | .. | + |
| M | 63 | N | 2 | | |
| F | 50 | C | 10 | .. | + |
| M | 61 | C | 3 | | |
| M | 59 | N | 4 | .. | .. |
| M | 60 | C | 5 | | |
| M | 57 | N | 13 | + | .. |
| M | 60 | C | 13 | | |
| F | 49 | N | 10 | .. | .. |
| M | 55 | N | 4 | | |
| F | 55 | C | 10 | .. | + |
| M | 62 | C | 3 | | |
| M | 58 | N | 3 | .. | .. |
| M | 58 | C | 4 | | |
| M | 55 | N | 4 | .. | .. |
| M | 58 | N | 6 | | |
| M | 64 | C | 2 | + | .. |
| M | 55 | C | 2 | | |
| M | 67 | N | 4 | .. | .. |
| M | 57 | C | 5 | | |
| M | 61 | N | 12 | + | .. |
| M | 60 | C | 13 | | |
| F | 49 | N | 10 | .. | .. |
| M | 61 | C | 3 | | |
| M | 59 | N | 4 | .. | .. |
| M | 55 | N | 4 | | |
| F | 55 | C | 10 | .. | + |
| M | 60 | C | 3 | | |
| M | 57 | N | 13 | + | .. |
| M | 49 | N | 4 | | |
| M | 54 | C | 10 | .. | + |
| M | 57 | C | 4 | | |
| M | 61 | N | 12 | + | .. |
| M | 58 | C | 5 | | |
| M | 60 | N | 10 | + | .. |

FIG. 1.—The graph for sequential analysis of the data. The slopes AB, AC, DE and FG were calculated by the methods of Armitage (1960) for an open design having a two-sided significance level $2\alpha = 0{\cdot}05$. The sample path is the zig-zag line starting at O and moving one unit diagonally upwards for each chronofusor preference and one unit diagonally downwards for each nitrogen mustard preference. Since the sample path enters the area enclosed by the lines AB and AC the difference between the two treatments is not statistically significant at the 5% level.

lines AB and AC which means that under the experimental conditions already defined the difference between the two treatments is not statistically significant at the 5% level.

### DISCUSSION

The aptness of sequential analysis for clinical trials of therapies depends upon the serial entry of patients to the trial and the continuous scrutiny of the results. These are two conditions occurring naturally in medical practice. In addition, the ethical demand for the most effective treatment can often be met at an earlier stage of assessing observations as they become available, rather than by awaiting the completion of a trial involving a predetermined number of patients. Thus the burden of our ignorance is inflicted upon the smallest number of patients and the unnecessary use of inferior treatments is avoided.

Cancer of the lung was studied for three reasons:

(1) These tumours are infrequently resectable and in general poorly radiosensitive, thus there is a need for the development of other forms of treatment, and this is an ever-increasing need as judged by the rising incidence of the disease, despite the commonly-known indictment of cigarette smoking.

(2) There are a number of favourable reports of intravenous chemotherapy (Galton, 1958; Karnofsky, 1959; Goldman, 1963; Barran, Helm and King, 1965).

Bronchial arterial infusion chemotherapy has also been effective but it is unlikely that prolonged infusion by this route would be widely applicable. In two studies (Kahn, Paul and Rheinlander, 1965; Wirtanen and Ansfield, 1968) only one-quarter of the attempts to achieve selective bronchial arterial infusion were successful and infusions could not be maintained longer than two to fifteen days. Unwanted effects may be associated with bronchial arterial infusion according to reports of para-oesophageal, intercostal and spinal anastomoses by Viamonte, Parks and Smoak (1965). The same detailed radiological study and other anatomical dissections (Wright, 1938) demonstrate the unpredictability of both bronchial and pulmonary vascularisation of primary pulmonary neoplasms and the frequency with which more than one bronchial artery may supply each lung. Intravenous chemotherapy was chosen for this study because it is applicable to all patients. The naturally high concentration of an intravenous infusate in the pulmonary circulation may be advantageous.

(3) The poor prognosis in cancer of the lung means that a proportionately large prolongation of life is obvious at a relatively early time of assessment such as six months after starting the treatment of each patient.

Although the assessment of the " vital " value of constant prolonged chemotherapy is negative there are a number of positive aspects to this trial. The applicability of the general principles of statistical control to therapeutic trials in cancer of the lung, and the specific value of Armitage's Sequential Analysis, have been demonstrated. It seems that this method could usefully form the basis of future assessments of new therapeutic regimes. The highest standards of patient care have not been sacrificed upon the altar of science, on the contrary they may have been enhanced by the emotional benefits felt even by intelligent patients reassured by the therapeutic activity and the interest in their problems. This assessment can be readily dismissed by the scientific purist as " only psychological " and not real in terms of prolonged survival, but this type of psychological support is a reality to the patient. Any simple technique of drug administration can provide support that encouraging words alone cannot give.

This trial has not shown that either regime gives a longer survival than the natural course of the disease determines. A useful comparison is with patients receiving palliative treatment in the form of radiotherapy, chemotherapy or surgery. The Cancer Registration Bureau of the Western Regional Hospital Board in Scotland published suitable data (1967). The twelve-month survival for these palliative treatments in the triennium 1962–64 was 15·2% and for untreated patients it was 13·9%, whereas the overall twelve-month survival rate in the series of patients described herein is 24·9%. Comparison of these two groups is not statistically valid but if the view that placebo or no-treatment groups are ethically inadmissible in cancer is held then no other assessment of this sort is available.

There is a widespread resistance amongst doctors to accept that it is more ethical to apply new treatments randomly according to a judicious protocol than to treat a selected series of patients. This attitude has one of two possible results. Either an effect is not demonstrated and the treatment is discarded, or non-randomised methods are continued because there has been apparent benefit and it is thought unethical to deprive future patients of a possibly beneficial treatment. The excuse that impossibly large numbers of patients are required to allow definitive conclusions to be drawn is often made. Many trials, including that reported in this paper, show this to be untrue, and such an argument could more forcibly be applied to those who make judgements upon large uncontrolled series of patients. This unwillingness to apply the principles of clinical measurement more widely has no easy solution, meanwhile it is clear that the necessitous field of cancer therapy requires new regimes and adequate evaluation of them in trials designed to answer specific questions.

### SUMMARY

A controlled clinical comparison of two methods of chemotherapy for cancer of the lung has been assessed by the sequential method of Armitage. No difference at the 5% level of statistical significance, in terms of patient survival, exists between the two treatments. It is concluded that this type of clinical measurement of treatments for cancer does not jeopardise standards of patient care and that it forms a sound basis for the assessment of new treatments.

A grant from the British Empire Cancer Campaign for Research to Mr. J. Maxwell Anderson made this trial possible. Further support came from the Board of Management of Glasgow Royal Infirmary, the Western Regional Hospital Board and Cancer International Research Cooperative.

We thank Mr. R. A. Elton, lecturer in statistics at Glasgow University, for statistical advice, our colleagues who referred patients for treatment, and Sister E. Callaghan for assistance.

We are also grateful to Sandoz Products Limited for supplies of SPI.

### REFERENCES

ANDERSON, J. M.—(1966) Paper presented at the 7th Annual Meeting of the British Association for Cancer Research.
ANDERSON, J. M., SMITH, M. D. AND HUTCHISON, J.—(1966) *Br. med. J.*, ii, 641.
ANDERSON, J. M. AND HUTCHISON, J.—(1968) *Dis. Chest*, **53**, 551.
ARMITAGE, P.—(1960) 'Sequential Medical Trials.' Oxford (Blackwell).
BARRAN, K. M., HELM, W. H. AND KING, D. A. R.—(1965) *Br. med. J.*, ii, 685.
GALTON, D. A. G.—(1958) 'Carcinoma of the Lung.' Edinburgh (Livingstone).
GOLDMAN, K. P.—(1963) *Br. med. J.*, i, 312.
KAHN, P. C., PAUL, R. E. AND RHEINLANDER, H. F.—(1965) *J. thorac. cardiovasc. Surg.*, **50**, 640.
KARNOFSKY, D. A.—(1959) *Ann. N.Y. Acad. Sci.*, **68**, 899.
LEES, A. W.—(1965) *Br. med. J.*, i, 230.
VIAMONTE, M., PARKS, R. E. AND SMOAK, W. M.—(1965) *Radiology*, **85**, 205.
WIRTANEN, G. W. AND ANSFIELD, F. J.—(1968) *Cancer Chemother. Rep.*, **52**, 263.
WRIGHT, R. D.—(1938) *J. Path. Bact.*, **47**, 489.
Western Regional Hospital Board of Scotland, Cancer Registration Bureau. Progress Note No. 7. 1967.